# Supplementary material for: Effect of Seminal Plasma on the Freezability of Boar Sperm
Source: Animals (Basel). 2024 Dec 18;14(24):3656. doi: 10.3390/ani14243656 (PMC11672632; doi:10.3390/ani14243656)
Supplement: Supplementary file 1 [file animals-14-03656-s001.zip › animals-3285210-supplementary.pdf]

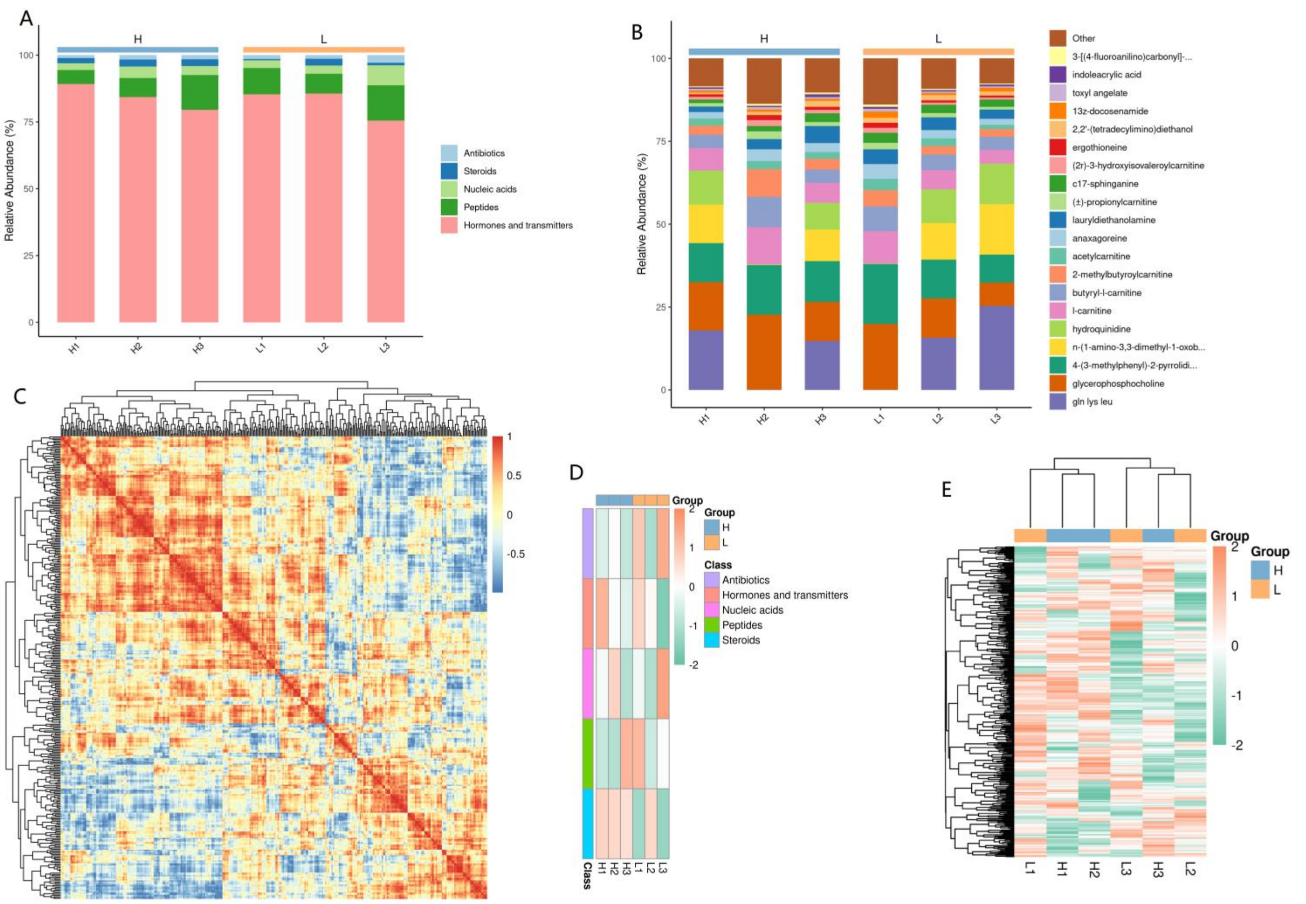

Supplemental Figure S1. metabolic compounds analysis of seminal plasma. A, relative abundance of seminal plasma metabolic compounds in goup H and L by type of compounds. B, relative abundance top 20 of seminal plasma metabolic compounds in goup H and L. C, relativity heat map of compounds in seminal plasma. D, C, relativity heat map of compounds type in seminal plasma. E, cluster map of compounds in different samples. H, high freezability goup; L, low freezability goup. H1,H2,H3, samples of group H; L1, L2, L3, samples of group L.

Table S1. Materials of metabolomic experiments

Reagent name	Supplier	Reagent name	Supplier
Dibasic Sodium Phosphate	Aladdin	Dimethylbenzene	Sangon Biotech
Sodium dodecyl sulfate, SDS	Amresco	Ammonium persulfate, APS	
Glycine		Agarose gel	
Ponceau S		Glycerol	
Milk powder		Triton X-100	
BCA Kit	Beyotime Biotechnology	Urea	Sigma Aldrich
Coomassie brilliant blue		Iodoacetamide, IAM	
ProteoMiner™ Protein Enrichment Small-Capacity Kit	Bio-Rad Laboratories	Tetraethylammonium bromide, TEAB	
Nitrocellulose filter membrane, NC		Trichloromethane	
Polyvinylidene fluoride, PVDF		Acrylamide	
Ultrapure water ,H_2O	Fisher Chemical	Trichloroacetic acid,TCA	
Formic acid	Fluka	Trifluoroacetic acid, TFA	
2-D Quant Kit	GE Healthcare	β-mercaptoethanol	
Ethanol	Hangzhou Gaojing Fine Chemical Industry	Ammonium bicarbonate	
Acetic Acid		Ethylene Diamine Tetraacetic Acid, EDTA	
Acetone	Hangzhou Hannuo Chemical	N,N,N,N-Tetramethylethylenediamine,TEMED	
Trichostatin A ， TSA	MedChemExpress	Tris-base	
Protease Inhibitor Cocktail III	Merck Millipore	Nicotinamide, NAM	
Protease Inhibitor Cocktail V		Hydrochloric acid	
Protease Inhibitor Cocktail VI		DL-Dithiothreitol, DTT	
Protease Inhibitor Cocktail IV		Sodium chloride	
Phosphorylase inhibitor	Millipore	Potassium chloride	
Horseradish Peroxidase, HRP		Potassium dihydrogen phosphate	
Mouse IgG (H+L) Secondary Antibody	Pierce	Seppro ® Rat Spin Columns	
Rabbit IgG (H+L) Secondary Antibody		Ammonium acetate	Sinopharm Group
Pierce Top 12 Abundant Protein Depletion Spin Columns	Thermo scientific	Phenol reagent for DNA extraction	
Protein Marker		D(+)-Sucrose	Solarbio
Acetonitrile	ThermoFisher Scientific	PAGE Silver dye kit	
Methanol		Trypsin	Promega
TMT label reagent		PR-619	Selleck Chemicals

Table S2. metabolites associated with the mean PM of 10min and 2h after thawing

Index	Compounds	H/L FC	H/L P value	H/L VIP	*r*	*p*
PTM_507	Myristamine Oxide	0.61	0.131	1.52	-0.958	0.003
PTM_505	Minoxidil	0.83	0.054	1.74	-0.947	0.004
PTM_329	C17-Sphinganine	0.67	0.121	1.53	-0.883	0.020
PTM_573	Octenoyl-carnitine	0.72	0.124	1.57	-0.875	0.022
PTM_117	2-Hydroxy-4-(octyloxyl)benzophenone	0.67	0.244	1.28	-0.827	0.042
PTM_784	Spisulosine	0.80	0.256	1.22	-0.826	0.043
PTM_116	2-ethylsulfanyl-N-[[1-(hydroxymethyl)cyclopropyl]methyl]benzamide	0.57	0.014	1.98	-0.818	0.047
PTM_86	2-(3,4-Dihydroxyphenyl)-5-hydroxy-7-methoxy-4-oxo-4H-chromen-3-yl 2-O-beta-D-xylopyranosyl-beta-D-glucopyranoside	1.72	0.246	1.23	0.812	0.050
PTM_45	1-Palmitoyl-2-lauroyl-sn-glycero-3-phosphorylcholine	1.67	0.035	1.85	0.815	0.048
PTM_198	4-Dodecylmorpholine	1.25	0.219	1.26	0.817	0.047
PTM_69	1-Stearoyl-2-hydroxy-sn-glycero-3-phosphoethanolamine	1.16	0.278	1.15	0.820	0.046
PTM_796	Tromethamine	1.44	0.155	1.48	0.830	0.041
PTM_506	MMV153413	1.18	0.272	1.17	0.833	0.040
PTM_709	Phthalic anhydride	1.25	0.311	1.09	0.836	0.038
PTM_402	Gly Gly Gln	1.25	0.298	1.11	0.838	0.037
PTM_606	PE 19:1	1.91	0.001	2.11	0.839	0.037
PTM_282	Asp Asp Tyr	1.80	0.016	1.94	0.840	0.036
PTM_349	Creatine	1.39	0.074	1.68	0.845	0.034
PTM_182	3-Oxo-1,8-octanedicarboxylic acid	1.15	0.216	1.26	0.847	0.033
PTM_406	Glycerophosphocholine	1.32	0.294	1.12	0.847	0.033
PTM_7	(2S,3S)-3,5,7-trihydroxy-6-methyl-2-(3,4,5-trihydroxyphenyl)-2,3-dihydrochromen-4-one	1.25	0.254	1.21	0.866	0.026
PTM_739	PS 34:2	7.12	0.018	1.94	0.875	0.022
PTM_397	Genistein	1.53	0.132	1.51	0.878	0.021
PTM_608	PE 21:2	1.21	0.080	1.61	0.889	0.018
PTM_754	S-Adenosylhomocysteine	1.44	0.055	1.72	0.890	0.017
PTM_248	5-[(Benzylsulfanyl)methyl]-2-furoic acid	1.18	0.086	1.64	0.914	0.011
PTM_166	3,4-Dihydroxybenzaldehyde	1.19	0.128	1.52	0.915	0.011
PTM_552	N-cyclopentyl-3-[4-(5-cyclopropyl-1,2,4-oxadiazol-3-yl)phenoxy]pyrrolidine-1-carboxamide	2.74	0.026	1.85	0.917	0.010
PTM_336	CAY10606	1.48	0.002	2.06	0.918	0.010
PTM_457	Leu Pro Asp	1.24	0.189	1.36	0.921	0.009
PTM_567	N-Tetracosanoyl-4-sphingenyl-1-O-phosphorylcholine	8.77	0.032	1.85	0.929	0.007
PTM_715	POV-PC	1.41	0.141	1.49	0.929	0.007
PTM_485	Lys Asp Pro Ile	1.81	0.175	1.40	0.933	0.007
PTM_610	PE 21:4	2.73	0.028	1.91	0.803	0.055
PTM_159	2-Propenal, 3-(1,3-benzodioxol-5-yl)-	1.26	0.365	1.02	0.803	0.055
PTM_80	2,2-Bis(hydroxymethyl)-3-quinuclidinone	1.18	0.132	1.43	0.806	0.053
PTM_442	L- Carnitine	1.26	0.291	1.11	0.810	0.051

Table S3. DEPs（FC>1.5） between highly and lowly freezable SP

Protein accession	Gene name	Protein name	H/L FC	H/L P value
P02475	CRYAA	Crystallin αA	2.45	0.005
A0A286ZTX1	CUTC	Copper homeostasis protein cutC homolog	2.39	0.030
A0A8W4FA46	SHANK1	SH3 and multiple ankyrin repeat domains 1	2.28	0.012
A0A5G2R9A9	PFN1	Profibrin 1	2.25	0.016
A0A286ZKF8	ENPP2	Ectonucleotide pyrophosphatase	2.02	0.042
A0A4X1T7K7	PLS1	Plastin 1	1.97	0.009
A0A4X1SM39	CRYBB2	Crystallin βB2	1.95	0.009
I3LC61	CYLC2	Cylicin 2	1.92	0.001
A0A8W4FAP8	HIP1	Huntingtin interacting protein 1	1.86	0.038
A0A287B3B2	PLA1A	Phospholipase A1 member A	1.80	0.042
F1S881	LOC100626120	Ig-like domain-containing protein	1.78	0.032
F2Z5N0	PSMA6	Proteasome 20S subunit alpha 6	1.72	0.049
Q007T1	CRYBB1	CrystallinβB1	1.71	0.021
A0A287AL10	FTH1	Ferritin heavy chain 1	1.71	0.033
Q29290	CSTB	Cathepsin B	1.71	0.040
A0A5G2RAG2	DSTN	Destrin, actin depolymerizing factor	1.61	0.032
A0A5G2R5Y6	CCT6A	Chaperonin containing TCP1 subunit 6A	1.57	0.024
A0A287BDX3	FURIN	Furin	1.56	0.016
A0A287AEA2	PPIC	Peptidylprolyl isomerase C	1.52	0.044
A0A287A391	EEF1A1	Eukaryotic translation elongation factor1α1	1.51	0.020
A0A8D0Z570	ACYP1	Acylphosphatase 1	0.66	0.0005
A0A8D1ZB16	TNFRSF21	TNF receptor superfamily member 21	0.63	0.0384
A0A5G2RD35	ATP6AP1	ATPase H^+ transporting accessory protein 1	0.62	0.0468
A0A286ZX63	GPRC5C	G protein-coupled receptor class C group 5 member C	0.62	0.0167
A0A287AN07	PTHLH	Parathyroid hormone like hormone	0.60	0.0130
A0A5G2QZ26	EPCAM	Epithelial cell adhesion molecule	0.60	0.0446
P48819	VTN	Vitronectin	0.57	0.0348
A0A5G2RAW2	GALNT18	Polypeptide N-acetylgalactosaminyltransferase 18	0.55	0.0081
A0A286ZQK6	ZG16B	Zymogen granule protein 16B	0.54	0.0338
K7GPY3	EDA	Ectodysplasin A	0.53	0.0108
A0A287AR98	SBSN	Suprabasin	0.50	0.0296
A0A287ACN2	ADIRF	Adipogenesis regulatory factor	0.48	0.0320
A0A4X1U0W1	CNOT7	CCR4-NOT transcription complex subunit 7	0.48	0.0343
A0A5G2RH94	NEU1	Neuraminidase 1	0.40	0.0229
A0A5G2QFI9	SAA3	Serum amyloid A-3 protein	0.37	0.0137
F1S7A4	TACSTD2	Tumor associated calcium signal transducer 2	0.33	0.021
A0A481BCM9	APOA2	Apolipoprotein A2	0.25	0.038
A0A287BKA9	CCN6	Cellular communication network factor 6	0.24	0.010

DEPs, differencial expressed proteins. FC, fold_change. SP, seminal plasma.

Table S4. PAMPs also DEPs between highly and lowly freezable seminal plasma

Protein accession	Gene name	Protein name	H/L		Pearson correlation	
			FC	P value	*r*	*p*
A0A5G2R599	FUCA2	α -L-Fucinosidase 2	1.21	0.003	0.915	0.01
P02475	CRYAA	crystallin alpha A	2.45	0.005	0.924	0.008
A0A4X1SM39	CRYBB2	Crystallin beta B2	1.95	0.009	0.841	0.036
A0A4X1T7K7	PLS1	Plastin 1	1.97	0.009	0.885	0.019
A0A287BDX3	FURIN	Furin	1.56	0.016	0.879	0.021
A0A5G2R9A9	PFN1	Profilin-1	2.25	0.016	0.922	0.009
A0A287A391	EEF1A1	Eukaryotic Translation Elongation Factor 1 Alpha 1	1.51	0.02	0.943	0.005
Q007T1	CRYBB1	Crystallin beta B2	1.71	0.021	0.925	0.008
P31346	ANG	Angiogenin	1.27	0.027	0.915	0.011
A0A287AW67	LGMN	Legumain	1.22	0.027	0.866	0.026
A0A4X1TEM7	PKM	Pyruvate kinase M	1.29	0.028	0.917	0.01
A0A287AV20	NAGA	N-acetyl galactosamine	1.21	0.029	0.922	0.009
F1S881	LOC100626120	Ig-like domain-containing protein	1.78	0.032	0.908	0.012
A0A5G2QSI4	IL1R1	Interleukin-1 receptor-1	1.27	0.035	0.981	0.001
A0A287A770	NPTN	Neuroplastin	1.47	0.037	0.958	0.003
A0A286ZKF8	ENPP2	Ectonucleotide pyrophosphatase	2.02	0.042	0.87	0.024
A0A5G2QSK0	PRSS8	Serine protease 8	1.26	0.05	0.849	0.033
A0A8D0Z570	ACYP1	Acylphosphatase 1	0.66	0	-0.828	0.042
A0A2C9F3B0	CTSS	Cathepsin S	0.77	0.008	-0.837	0.038
A0A5G2RAW2	GALNT18	Polypeptide N-acetylgalactosaminyltransferase 18	0.55	0.008	-0.886	0.019
A0A287BKA9	CCN6	Cellular communication network factor 6	0.24	0.01	-0.961	0.009
K7GPY3	EDA	Ectodysplasin A	0.53	0.011	-0.889	0.018
A0A5G2QFI9	SAA3	Serum amyloid A-3 protein	0.37	0.014	-0.954	0.012
A0A480K168	--		0.84	0.017	-0.962	0.002
A0A286ZX63	GPRC5C	G protein-coupled receptor class C group 5 member C	0.62	0.017	-0.869	0.025
A0A5G2RH94	NEU1	Neuraminidase 1	0.4	0.023	-0.925	0.024
A0A287AR98	SBSN	Suprabasin	0.5	0.03	-0.815	0.048
A0A286ZQK6	ZG16B	Zymogen granule protein 16B	0.54	0.034	-0.849	0.032
A0A4X1U0W1	CNOT7	CCR4-NOT transcription complex subunit 7	0.48	0.034	-0.981	0.001

PAMPs, proteins associated with mean PM(progress motility) of 10min and 2h after thawing. DEPs, differencial expressed proteins. FC, fold_change. *r*, pearson correlation coefficient. *p*, P value of *r*.

Table S5. PAMPs but not DEPs between highly and lowly freezable SP

Protein accession	Gene name	Protein name	H/L		Pearson correlation	
			FC	P value	*r*	*p*
A0A5G2R8P4	PCDHAC2	Protocadherin alpha subfamily C, 2	2.94	0.153	0.979	0.021
A0A287A5G1	ACTG1	Actin, cytoplasmic 2	1.18	0.087	0.962	0.002
A0A287B988	ADAM32	A Disintegrin and Metalloproteinase Domain-32	1.32	0.124	0.959	0.003
Q8SPJ0	RNASE10	Inactive ribonuclease-like protein 10	2.30	0.076	0.948	0.004
A0A287B9B4	DEFB113	Beta-defensin113	1.80	0.148	0.942	0.005
A0A4X1W2B1	TPM3	Tropomyosin 3	1.21	0.154	0.941	0.005
A0A287BN95	DEFB128	Beta-defensin128	1.60	0.129	0.930	0.007
A0A5S8KLN1	CLU	Clusterin	2.16	0.052	0.926	0.008
A0A8D0YLA6	--	Beta-defensin	1.99	0.138	0.919	0.010
A0A287A191	DSC2	Desmocollin 2	1.27	0.139	0.917	0.010
Q1RLJ4	DEFB129	Beta-defensin129	1.24	0.173	0.906	0.013
A0A287AE72	TEX101	Testis expressed101	1.48	0.212	0.897	0.015
A0A287AAI1	CLEC11A	C-type lectin domain containing 11A	1.74	0.228	0.893	0.041
A0A5G2R207	GOLM2	Golgi membrane protein 2	1.39	0.237	0.891	0.017
F1REZ1	HAPLN1	Hyaluronan and proteoglycan link protein 1	2.01	0.129	0.886	0.019
A0A5G2R7E3	FAM3D	Family with sequence similarity 3,member D	1.18	0.181	0.886	0.019
A0A287AE71	LCN9	Lipocalin-9	1.67	0.261	0.876	0.022
A0A287A5R7	PRSS55	Serine protease-55	1.52	0.185	0.875	0.023
A0A8D0QE71	CRYGS	Crystallin γS	1.53	0.098	0.869	0.025
A0A287ALA1	SERPINA1	Serpin family A member 1	1.88	0.054	0.867	0.025
A0A287BL44	ADAM7	A Disintegrin and Metalloproteinase Domain-7	1.33	0.286	0.866	0.026
F1SGP8	RCN1	Reticulocalbin-1	1.35	0.144	0.860	0.028
A0A287B9B7	LCN15	Lipocalin-15	1.54	0.280	0.859	0.028
A0A286ZW80	CENPE	Centromere protein E	1.57	0.294	0.850	0.032
A0A4X1W4T1	LYPD4	LY6/PLAUR domain containing 4	1.73	0.212	0.849	0.033
A0A286ZX86	CES5A	Carboxylic ester hydrolase 5A	1.32	0.260	0.844	0.035
A0A287BLV6	BPI	Bactericidal permeability-increasing protein	3.39	0.223	0.841	0.036
A0A5K1UHA8	ART3	NAD(P)(+)--arginine ADP-ribosyltransferase3	1.46	0.210	0.838	0.037
A0A287BBM3	GML	glycosylphosphatidylinositol anchored molecule like protein	1.74	0.127	0.836	0.038
A0A287AHL2	SEZ6L	Seizure related 6 homolog like	2.75	0.329	0.834	0.039
A0A4X1T2C1	QPCT	Glutaminyl-peptide cyclotransferase	1.27	0.226	0.830	0.041
F1S232	ALDH9A1	aldehyde dehydrogenase 9 family member A1	1.27	0.056	0.827	0.043
A1IU54	CPE	Carboxypeptidase E	1.24	0.300	0.820	0.045
F1SNJ4	CREB3L2	cAMP responsive element binding protein 3 like 2	0.53	0.145	-0.821	0.045
O46420	CYP51A1	Lanosterol 14-alpha demethylase	0.61	0.076	-0.839	0.037
F1RRJ3	YIPF3	Yip1 domain family member 3	0.76	0.077	-0.848	0.033
Q8MI70	E3	Epididymal protein 3B	0.70	0.157	-0.870	0.024
A0A287A3E9	SEMA3F	Semaphorin 3F	0.79	0.087	-0.897	0.015
A0A5G2QYS9	AFM	Afamin	0.61	0.252	-0.965	0.035
I3L7Z6	S100A6	S100 calcium binding protein A6	0.60	0.065	-0.970	0.001
F1RXG1	KRT27	Keratin, type I cytoskeletal 27	0.35	0.363	-0.994	0.006

PAMPs, proteins associated with mean PM(progress motility) of 10min and 2h after thawing. DEPs, differencial expressed proteins. SP, seminal plasma. FC, fold_change. *r*, pearson correlation coefficient. *p*, P value of *r*.
